# The Pathology of the Brain Eating Amoeba *Naegleria fowleri*

**DOI:** 10.1007/s12088-024-01218-5

**Published:** 2024-03-06

**Authors:** Yannick Borkens

**Affiliations:** 1 Institut für Pathologie, Charité Campus Mitte, Virchowweg 15, Charité, 10117 Berlin, Germany; 2 Humboldt-Universität zu Berlin, Unter den Linden 6, 10117 Berlin, Germany

**Keywords:** Amoebic, Nasal infections, Parasites, Protozoa, Tropical medicine

## Abstract

The genus *Naegleria* is a taxonomic subfamily consisting of 47 free-living amoebae. The genus can be found in warm aqueous or soil habitats worldwide. The species *Naegleria fowleri* is probably the best-known species of this genus. As a facultative parasite, the protist is not dependent on hosts to complete its life cycle. However, it can infect humans by entering the nose during water contact, such as swimming, and travel along the olfactory nerve to the brain. There it causes a purulent meningitis (primary amoebic meningoencephalitis or PAME). Symptoms are severe and death usually occurs within the first week. PAME is a frightening infectious disease for which there is neither a proven cure nor a vaccine. In order to contain the disease and give patients any chance to survival, action must be taken quickly. A rapid diagnosis is therefore crucial. PAME is diagnosed by the detection of amoebae in the liquor and later in the cerebrospinal fluid. For this purpose, CSF samples are cultured and stained and finally examined microscopically. Molecular techniques such as PCR or ELISA support the microscopic analysis and secure the diagnosis.

Infectious diseases continue to be a major cause of illness and death, especially in poorer countries of the global South [1]. The pathogens that cause these diseases are diverse and rich in form. Alongside viruses and bacteria, parasites represent the third major pathogen caste of disease-causing agents [2]. Parasites occur, for example, in the form of intestinal worms or unicellular eukaryotes, so-called protists. Many dangerous and relevant diseases are caused by such protists. For example malaria, sleeping sickness, or Chagas disease. Many are dangerous tropical diseases that are relevant to travellers of the temperate countries of the global west [3] (Fig. 1).

**Fig. 1:**
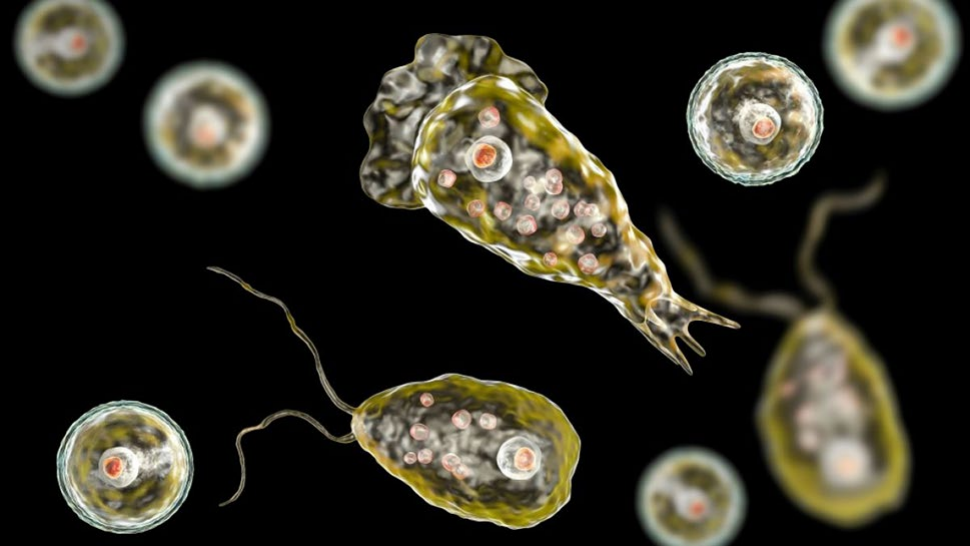
*Naegleria fowleri*

The systematic classification of the protists is unclear and is subject to regular changes and adaptations. It is clear that protists are not a systematic clade in the strict sense [4, 5]. In some cases, they are referred to as algae [6]. Due to this diversity of forms, a meaningful classification that is relevant for targeted science is not easy. One possible classification focuses on the type of movement. Different protists have evolved different modes of locomotion: with cirripedes, cilia, flagella or as amoebae with pseudopodia [7]. Since the classification, like other types of protist characterization, focuses purely on external features and morphologies, there is no relationship within these classifications. Pathogens can be found in all of these classifications. For example, the causative agents of sleeping sickness and Chagas disease are distinguished by their characteristic flagella [8] (Fig. 2).

**Fig. 2:**
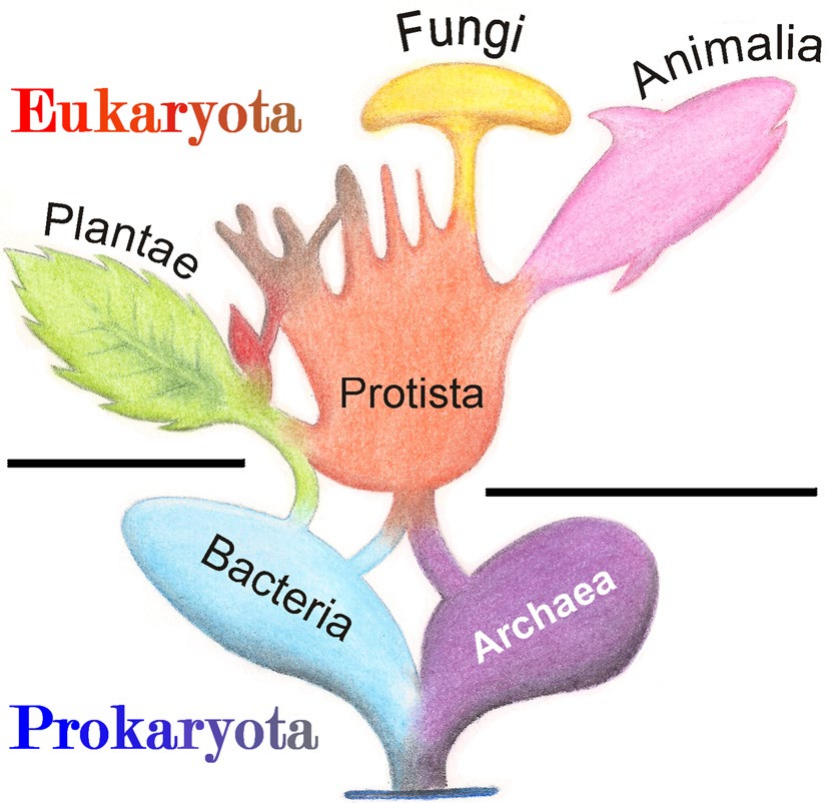
Simplified phylogenetic tree according to the 6-kingdom model of Woese et al. [109]. The cross-branches represent endosymbiosis events

This article deals with a different group of protists: the amoebae. Although amoebae may also cause dangerous diseases, they are overshadowed by other protist infections such as malaria, toxoplasmosis or Chagas disease.

Amoebae are also not a systematic clade, but a life form. Amoebae are unicellular organisms without a fixed body shape that are not closely related to each other. They move by the formation of so-called pseudopods. In doing so, the amoebae change their body shape [9]. At up to 1 mm, they belong to the largest protists. Some groups are shelled (*Thecamoebae*), but most are naked [10]. Amoebae feed heterotrophically via phagocytosis or autotrophically via photosynthesis. For this purpose, the autotrophic amoebae possess chloroplasts [11].

Amoebae are be found worldwide, including the Arctic and Antarctic [12]. Amoebae can also survive in airless space. For this purpose, they form cysts. However, most amoebae prefer moist soils and mud as well as water (fresh and salt water) [13]. The life form of the amoeba has evolved independently several times in evolutionary terms. In modern systematics, heterotrophic amoebae are usually classified as Amoebozoa, Rhizaria, and Excavata. Autotrophic forms usually belong to the group Chromalveolata [14, 15].

Many amoebae are pathogens for humans and can sometimes cause serious illnesses. One of the best known is probably amoebic dysentery. This is a severe gastrointestinal disease that causes, among other things, bloody, slimy diarrhea [16, 17]. Amoebic dysentery is caused by the entamoeba *Entamoebe histolytica* [18]. With approximately 50 million cases per year, amoebic dysentery is one of the most common protozoan infections [19–21]. Acanthamoebae can lead to severe inflammation of the cornea, especially in contact lens wearers [22]. For this reason, contact lenses should be cleaned regularly and the contact lens solution should also be changed regularly. Acanthamoeba keratitis is easy recognized and treat [23]. Amoebae can also cause inflammation in the oral cavity. *Entamoeba gingivalis* usually occurs in persistent gingivitis [24, 25]. In addition, amoebae can also serve as reservoir for pathogenic bacteria [26].

Much rarer than amoebic dysentery, but also much more extreme, is the so-called PAME. PAME stands for primary amoebic meningoencephalitis, a purulent inflammation of the brain. The associated pathogen is *Naegleria fowleri* [27].

*Naegleria fowleri* is a species of the genus *Naegleria*, which includes about 50 species. *Naegleria* belongs to the *Tetramitia*, the largest group of *Heterolobosea*, which groups together amoeboid protozoa. The *Heterolobosea* belong to the Excavata, one of the three eukaryotic supergroups [28, 29]. In this context, the Excavata are the only supergroup that is exclusively unicellular [30]. The remaining two, the Diaphoretickes and the Amorphea contain both unicellular and multicellular organisms. The Diaphoretickes include plants [31]. The Amorphea include fungi and animals [32]. The Excavata are divided into 7 groups without systematic rank: the *Fornicata*, the *Malawimonas*, the *Parabasalia*, the *Preaxostyla*, the *Jakobida*, the *Euglenozoa* and the *Heterolobosea* [30]. Species of the genus *Naegleria* are dependent on moisture and thus are found in moist soil as well as in stagnant water [33]. As it can spread optimally in warm waters, it can be found in swimming pools, bathing lakes and industrial wastewater [34]. Its distribution is not regionally limited. *N*. *fowleri* has been detected worldwide. Focal populations are found in the USA, Australia and France [35, 36]. Under ideal conditions, the trophozoites of *N*. *fowleri* form large colonies [37]. The trophozoite represents the amoeboid form. As a trophozoite, *N*. *fowleri* forms pseudopodia and feeds on bacteria and detritus. However, if the environmental parameters change, for example due to a drop in electrolyte levels, the cell forms flagella in order to escape quickly. If the parameters deteriorate further or escape is not possible, *N*. *fowleri* forms cysts. This cyst form is the smallest form with a size of up to 15 µm. The trophozoite is twice as large at 30 µm [38]. Figure 3 shows the different stages of the amoeba's life cycle.

**Fig. 3:**
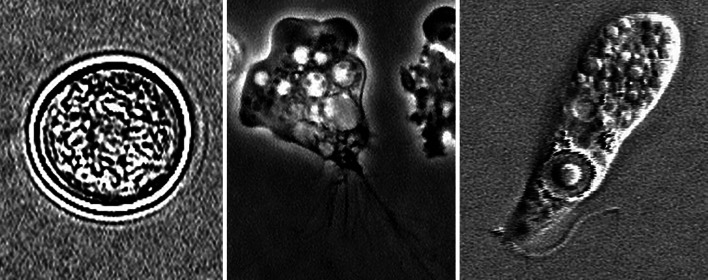
Forms of *Naegleria fowleri*. Left: Cyst; middle: Trophozoite; right: Flagellate

*Naegleria fowleri* is a so-called facultative parasite. This means that, unlike other parasites, the amoeba does not require a host for its life cycle. Like other *Naegleria* species, *N*. *fowleri* reproduces via mitosis [36].

*Naegleria* is interesting from a molecular point of view. This is because the cell is capable of transforming from an amoeboid form without a cytoskeleton to a flagellate form with complex cytoskeletal structures and flagella. The mechanism is the subject of research. *Naegleria gruberi* has been established as a model in this respect. Although *N*. *gruberi* is very closely related to *N*. *fowleri*, it is not a pathogen and thus its safe for researchers [39, 40]. *N*. *gruberi* is the only *Naegleria* species whose genome has been completely sequenced. Medicine could also benefit from this research. For example, the conversion from the amoeboid type to the flagellate type or the associated mechanism (including the de novo synthesis of centrioles) could be a theoretical target of therapeutics [41–43]. In practice, however, this is considerably complicated by the status of *N*. *fowleri* or PAME as a neglected disease [35]. In general, there is no therapeutic need for this disease, although indescribably tragic for the individual patient. The reason for this is the rarity of the cases.

## Route of infection

**Fig. 4:**
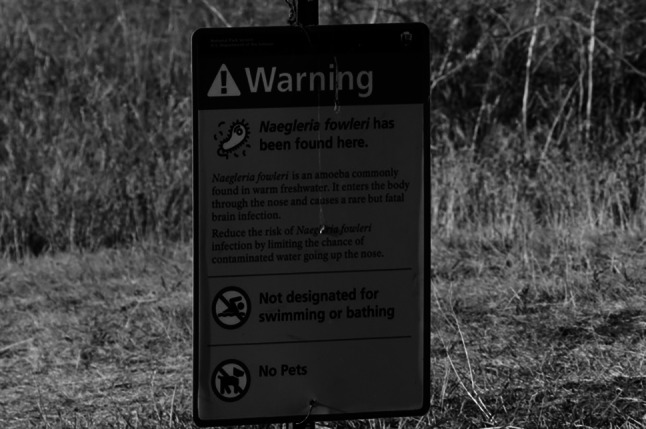
Warning sign from the United States. Alachua County, Florida

As already described, *N*. *fowleri* is a facultative parasite. This means that, unlike other protozoa such as *Plasmodium* spp. or *Toxoplasma gondii*, *N*. *fowleri* is not dependent on humans as hosts to complete its life cycle and thus reproduce [36, 44, 45]. Nevertheless, *N*. *fowleri* is capable of infecting humans and causing severe disease. In this case, infection occurs exclusively through the nose. If contaminated water is drawn up the nose, for example, during diving or when the head is held under water, *N*. *fowleri* cells can enter the brain via the olfactory nerve (*Nervus olfactorius*) [27, 36, 40]. Sources of infection are contaminated water sources such as lakes or swimming pools [37, 44] (see Fig. 4). Infections at home are also described less frequently. For example, via contaminated tap water [46, 47] or nasal rinsing [48]. This may also play a role in religious cleansing rituals [49, 50].

**Fig. 5:**
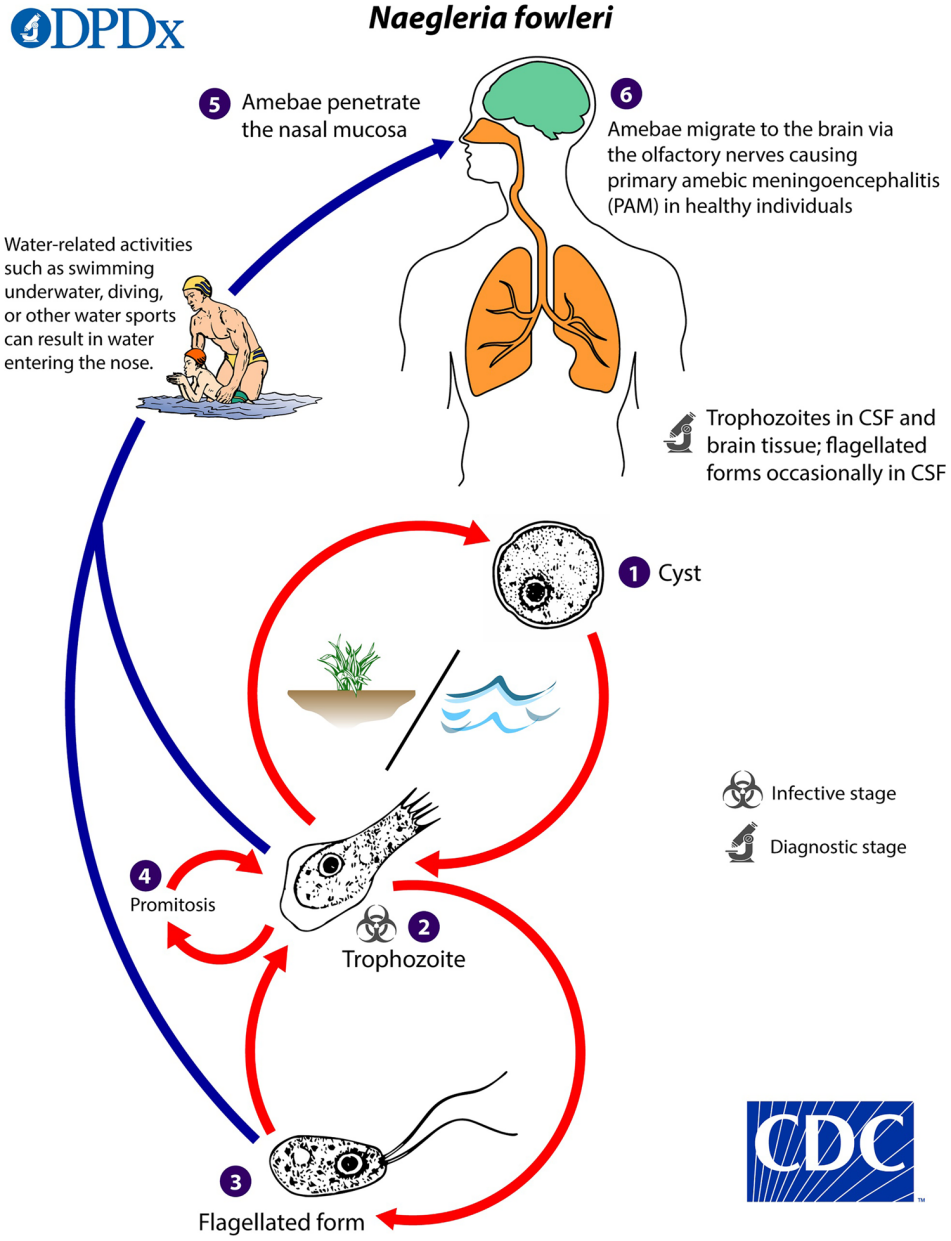
Life cycle of *Naegleria fowleri*. ©Centers for disease control and prevention, all rights reserved

*N*. *fowleri* cannot be ingested via the oral route. Ingestion of contaminated water is therefore not dangerous [51, 52]. Infections via water vapor or droplets have also not been reported [53, 54]. As* N*. *fowleri* is very resistant, treatment of water, for example, with chlorine, is only of limited help [55]. However, wearing nose clips while swimming can significantly reduce the risk [56, 57]. Transmission between humans is not possible. This includes organ transplants. These have been discussed as a possible route of transmission for some time. However, transmission through transplantation has not been confirmed [58, 59] (Fig. 5).

## Disease and Symptoms

If *N*. *fowleri* reaches the brain, the parasite causes a purulent meningitis. This meningitis is referred to in the clinic and in the literature as either primary amoebic meningoencephalitis (PAME or PAM), Naegleriasis or swimming pool amoebosis. The first symptoms usually appear between three and seven, but not later than 14 days, after exposure [45, 54]. The onset of symptoms is immediately and severe. At the beginning of the disease phase, PAME is characterized by severe nausea with vomiting, high fever, headache, and neck stiffness [60, 61]. The second phase is characterized by a pyogenic meningoencephalitis (the development of pus is called pyogenesis) and coma eventually occur [44, 45, 62]. Death occurs one week after the onset of symptoms. PAME mainly affects children and young adults. Therefore, similar to malaria, PAME can be considered a dangerous childhood disease [44, 62, 63]. However, infections with *Naegleria fowleri* are significantly rarer than with *Plasmodium* spp. About 400 cases are reported in the literature per year (381 PAM cases in 2018). Patients are usually male (75%) and have an average age of 14 years. In the US, 16 cases are reported for 2018, including 8 male and 8 female patients. All of these cases were fatal. PAME is considered extremely fatal. In the USA, of 157 documented cases between the years 1962 and 2022, only four patients survived [64–67]. *N*. *fowleri* is classified as a *neglected (tropical) disease* [35]. These NTDs include infectious diseases that are fatal and/or very dangerous, but not as focused on as other infectious diseases (neglected). The reason for this neglect can be manifold. For example, many neglected diseases play an important medical role in poorer countries of the global south. The health systems in these countries need to be expanded and are often dependent on donations from non-governmental organizations of development aid. As a result of this donation system, the financial resources required for research and treatment of these neglected diseases are often lacking. The lack of trained specialists is also a reason for this neglect [68]. *N*. *fowleri* is not reportable [66].

## Immunity

As there is still no reliable drug therapy against *N*. *fowleri*, understanding the immune response is important for research and the development of new drugs and therapeutics. As the infection is fatal in humans, immunologists rely on animal models.

### Innate Immune Response

Studies have shown that the infiltration of the amoeba cells in the nose occurs with little inflammation. This initial infiltration is in stark contrast to the aggressive inflammation that occurs later in the brain. The lethal inflammatory reaction ultimately results from the discovery and subsequent infiltration of immune cells (neutrophils, eosinophils, monocytes and macrophages) into the brain tissue [69]. The lack of an inflammatory response upon infiltration of the nasal tissue with subsequent strong reactions in the brain indicates that the amoebae have a way to bypass the innate immune response and invade the tissue undetected [70]. In contrast to bacterial and viral infections, the detection of protists by the immune system is more difficult. Since the cells of the pathogen are eukaryote just like the host cells, they are often recognized as non-foreign by the pattern recognition ability of the immune system [71]. Infections with amoebae can lead to increased activity of neutrophil granulocytes via antibody-mediated complement activation. However, studies show that *N*. *fowleri* have a certain resistance to the lysis mediated by this cascade. Only limited evidence shows that the complement system is relevant for immunology against *N*. *fowleri* [72].

In the host, *N*. *fowleri* feeds by trogocytosis, similar to how immune cells interrogate antigens from antige-presenting cells [73]. It can be assumed that this behavior puts the body cells under strong stress, which could, for example, lead to a strong release of substances like ATP [74]. However, there is little data on this approach. The situation is better when it comes to researching cytokines and their role in the immune response to *N*. *fowleri*. Tumor necrosis factor alpha (TNFα) probably plays an important role. This activates neutrophil cells, which in turn attack the amoebae. Important mechanisms of this attack are probably related to enzymes such as myeloperoxidase, superoxide formation or the release of so-called extracellular traps [75, 76]. Studies with animal models show that TNFα also plays a crucial role in the control of the disease and the development of PAM. Animals injected with TNFα did not develop PAM, even after the onset of the disease [77]. This knowledge is of particular importance for the development of new therapies. However, there are still many unanswered questions. For example, it is still unclear how the activated neutrophils detect the amoebae in the tissue and launch a coordinated attack. Individual neutrophil cells cannot tackle an *N*. *fowleri* amoeba with success [78].

### Innate Immune Response

The study of the adaptive immune response to *N*. *fowleri* infections is difficult due to the rapid lethal course. Surface-binding antibodies can be rapidly taken up by *N*. *fowleri* [79]. Thus, the amoeba can undermine the humoral immune response to a certain degree. However, since antibodies to a certain degree. However, since antibodies are constantly produced in vivo, this uptake is inhibited at some point. Immunization strategies experimenting with different antigens and cell states (amoeba lysate, living amoebae, fixed amoebae, specific proteins) showed that circulating antibodies are the main immune mechanism of the adaptive immune response [79]. The intrathecal administration of therapeutic monoclonal antibodies was able to prolong the survival of animal models. Antibodies affect *N*. *fowleri* in various ways. They can opsonize the cells and thus facilitate phagocytosis or effector activity. However, many antibodies against *N*. *fowleri* are directed against internal cell structures and do not target surface proteins [80]. During acute infection, IgM is produced by the immune cells. IgM is also used in infection with *N*. *fowleri* and can trigger agglutination and complement activation. However, IgM has difficulty to cross the blood–brain barrier due to its large molecular weight (approx. 900kD) [69].

In addition to the problem of the large molecular weight, other factors can negatively influence IgM. For example, various surface antigens of amoebic cells can promote T-independent reactions. Similar reactions can also be observed with certain bacterial polysaccharides [81]. Other factors that can favour an IgM bias are defects in the *Naegleria*-specific CD4 + repertoire of T Cells, defects in priming and defects in the functions that facilitate antibody class switching. In general, research into the innate immune response is still incomplete. Amoeba-specific CD4 + T cell functionality has bot been carefully studied for *N*. *fowleri* either after infections or for potential vaccines [82]. And although it has been observed that cell-mediated immunity against *N*. *fowleri* is associated with a time-delayed hypersensitivity, however, the need for research is still quite high. In animal models, it has been observed that IL-4 concentration is associated with animal survival. This may be relevant for vaccination research. As this effect is strongly dependent on STAT6 (Signal Transducer and Activator of Transcription 6), it can be assumed that Th2 cells play an important role in this process. STAT6 is essential for the signalling pathway that is responsible for the formation of Th2 cells and the associated immune response [83].

## Pathology and Diagnosis

In diagnostics, a fundamental distinction must be made between two temporal examinations: the premortem and the postmortem examination. The former serves the clinical diagnosis of the patient by using microscopic and molecular methods, the latter includes the autopsy and associated macroscopic examinations of the affected (brain) tissue [66].

Since the occurring symptoms are strong, but also quite unspecific. A precise diagnostic clarification is imperative. Pyogenic meningoencephalitides can have various causes, for example brain tumors or abscesses. Bacterial or viral encephalitides also cause almost identical symptoms. These conditions should be considered in the differential diagnosis. On the other hand, PAME should also be considered in cases of encephalitides that have not been further investigated (here, the travel history can also play an important role as a special part of the medical history). The diagnosis is made after microscopic and molecular examination. Molecular techniques include PCR and ELISA. There is a multiplex PCR that can be used to detect *Acanthamoebae*, *N*. *fowleri* and *B*. *mandrillaris* in combination [66, 84].

### Macroscopy

**Fig. 6:**
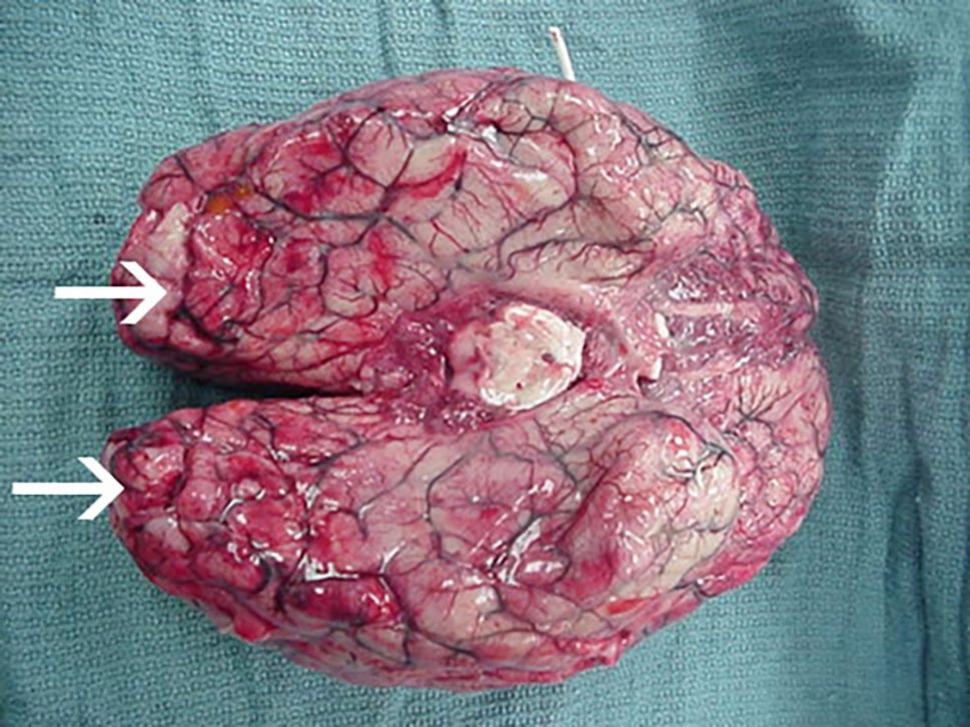
Infected brain from a PAM patient. The whole brain is shown. Extensive hemorrhage and necrosis is present, mainly in the frontal cortex. ©Centers for disease control and prevention, all rights reserved

**Fig. 7:**
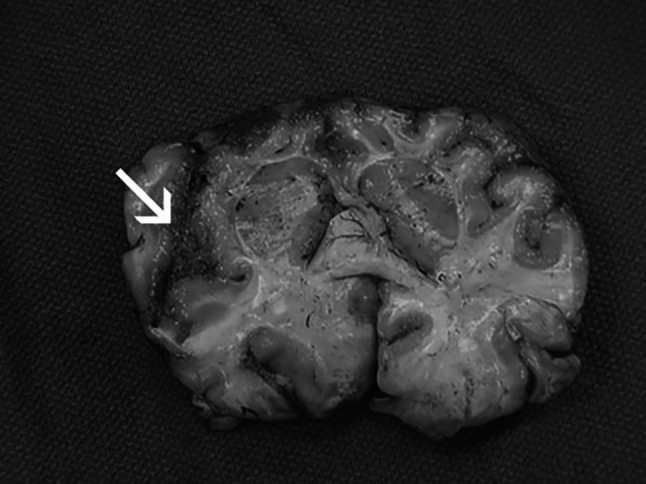
Infected brain from a PAM patient. A brain section is shown. Focal hemorrhage and necrosis in frontal cortex is visible. ©Centers for disease control and prevention, all rights reserved

Due to the aggressiveness and lethality of PAME, the pathological macroscopic examination can only be performed postmortem at the necropsy. This examination is primarily necessary to confirm or exclude “*Naegleria fowleri*/PAME” as the cause of death. The macroscopic examination is carried out by neuropathologists by examining the brain. PAME appears on the brain in the form of severe hemorrhages and associated necrosis of brain tissue. The hemorrhages are mainly localized in the frontal cortex. The Figs. 6 and 7 show an affected brain.  The tissue is typically further examined by microscopy and molecular biological methods [66].

### Microscopy and Histology

**Fig. 8:**
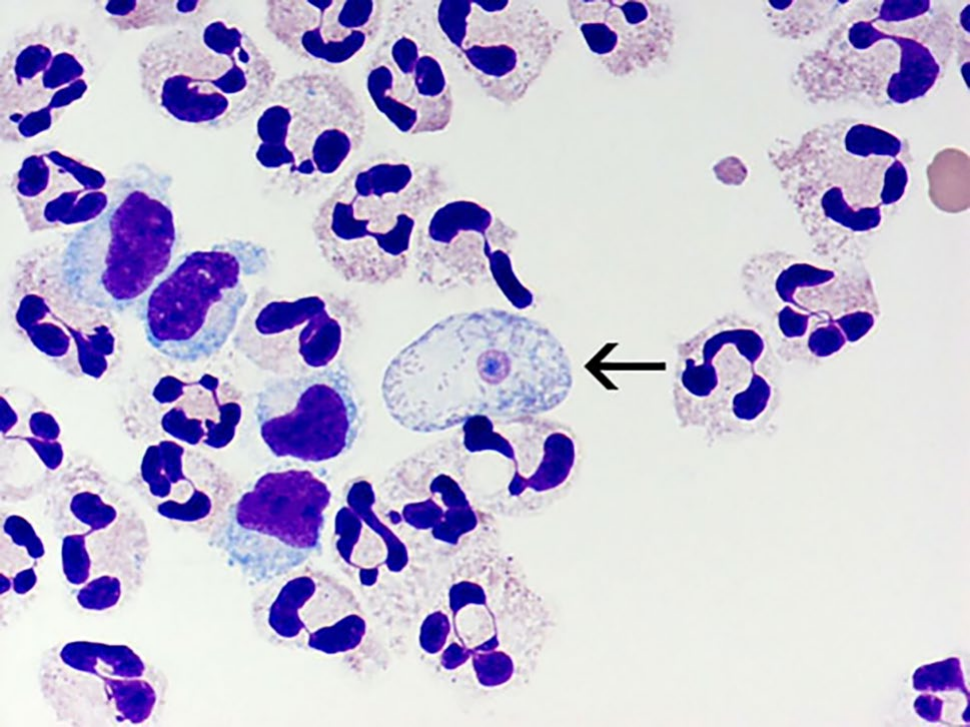
Cytospin of fixed CSF showing Giemsa-Wright stained trophozoite of *Naegleria fowleri* (arrow) amidst polymorphonuclear leukocytes and some lymphocytes. The nucleus and nucleolus can be seen within the trophozoite. Magnification: 1000x. ©Centers for disease control and prevention, all rights reserved

**Fig. 9:**
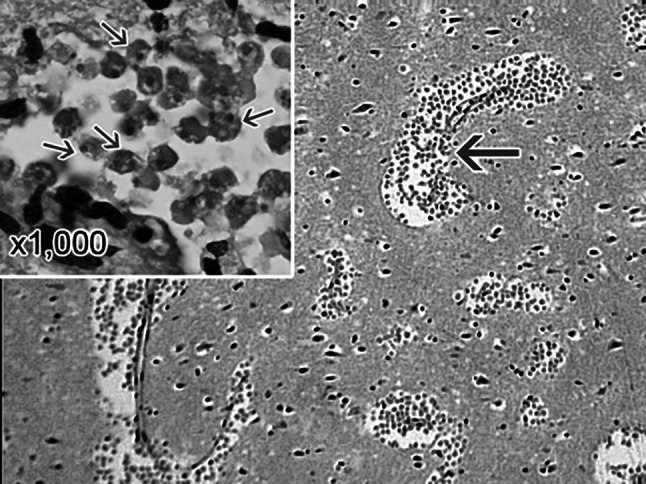
A section through the cerebral portion of the brain of a PAM patient stained with hematoxylin and eosin showing large accumulations of trophozoites of *Naegleria fowleri* and destruction of the normal architecture of the brain tissue. Cysts are not seen. Magnification: 100x. Inset: Higher magnification (1000x) of *Naegleria fowleri* trophozoites (arrows) showing characteristic nuclear morphology. ©Centers for disease control and prevention, all rights reserved

A microscopic examination is mandatory. As with other parasitic infections, it is the gold standard here and allows the detection of not only *N*. *fowleri*, but also other amoeba species. In this way, the diagnosis can be further narrowed down. Microscopic samples of the patients' cerebrospinal fluid are taken. These are obtained from the nose. A histological examination of the tissue of the Bulbus olfactorius is also permitted. The specimens can be stained. Examination after cultural cultivation is also common. Here it should be noted that the sample material is not frozen and is kept moist with a few drops of water. Otherwise, the amoebae could die. PAME is considered diagnosed when fast-moving sporozoites of *N*. *fowleri* are found. Molecular studies could confirm or further narrow down the species. A distinctive feature that can be exploited in the diagnosis of *N*. *fowleri* is its ability to differentiate cells. In hypotonic water, *N*. *fowleri* transforms into its flagellate form within two hours. This allows *N*. *fowleri* to be reliably diagnosed, as other pathogens do not have this ability [66, 85, 86] (see Figs. 8 and 9).

## Therapy

There is still no proven therapy that can be used for *N*. *fowleri*. Based on various laboratory studies and case studies, the Centers for Disease Control and Prevention recommends treatment with amphotericin B, which should be used in high doses. Amphotericin B is traditionally an antifungal agent used for severe fungal infections, but it is also used in the treatment of parasitic leishmaniasis. Amphotericin B should be administered intrathecally [66, 87–89].

Various studies examined the use of combined medications. In addition to amphotericin B, the drugs used in the studies also included fluconazole, chloramphenicol, dexamethasone, miconazole, rifampicin and miltefosine [90]. The success of the studies remained modest. A cure is very rarely. The studies showed that the effects are greatest when the drugs are administered early after exposure. The sooner the better. However, this is complicated by the rapid progression of the disease in conjunction with the diagnostic process. Another problem with the studies is that many effects have only described in vitro. Last but not least, the use in in vivo studies is ethically complex [90–92].

Miltefosine was used in some later cases [89, 93, 94]. It was shown that the success of the therapy was closely related to therapeutic temperature management (TTM) [95, 96]. The goal of TTM, also known as therapeutic hypothermia, is to lower and maintain a specific body temperature over a period of time [96]. Since *N*. *fowleri* attacks the brain, it is also important to pay attention to possible neurological damage during therapy. The effect of miltefosine on the central nervous system has also not been conclusively clarified. In 2013, a 12-year-old girl was successfully treated with miltefosine in combination with TTM. She survived the infection without neurological sequelae. However, the reason for this success was undoubtedly also the rapid diagnosis and intervention. At the same time, an 8-year-old boy was also treated with miltefosine. However, in his case TTM was omitted. He also survived, but with (probably) lifelong neurological damage. In 2016, the 12-year-old's therapy was successfully repeated in a 16-year-old male patient. This patient also survived without neurological damage. He himself simply states that learning has become more difficult for him in general [97].

In 2023, new results were published showing that treatment with benzoxaborole significantly improved life expectancy in infected mice and led to partial (28%) cure [98].

Recently, a new class of drugs and therapies has been emerging. As many anti-inflammatory agents are becoming outdated due to their sometimes severe side effects, new classes of agents are of particular interest [99]. These include the so-called anti-heterocyclic compounds. The synthesis of these active substances is accompanied by a number of advantages. Not only are they comparatively cheap to synthesize. They also have a wide range of pharmacological qualities. Studies have confirmed their antiviral, antibacterial, anticancer and insecticidal effects. Modern medical research also benefits from nanotechnology [100]. Silver nanoparticles (SNPs), for example, are of interest. In nanotechnology, materials are modified at atomic level. SNPs can be used to activate carbon composites. This is done by favourable photodeposition. This produces pure, well-defined SNPs. In addition to other uses, they play an important role as therapeutics agents. For example, as reagents and precursor for various formulations against COVID-19 or cancer. They can also be used as antioxidants [101, 102]. Novel substances such as these are also interesting for parasitology. Studies have already confirmed positive effects against malaria and other parasitic infections [103]. Drugs used against *N*. *fowleri* showed better effects when they were previously conjugated with silver nanoparticles. This was the case for nystatin and amphotericin B, but not for fluconazole. The researchers hope that this modification will enhance the effect of existing drugs to such an extent that the development of new drugs (which also involves investing a lot of money) will become at least partially obsolete [104].

It is questionable how likely the development of a vaccine against *N*. *fowleri* is. Due to the rarity of the disease, it is not the focus of profit-oriented pharmaceutical companies. Nevertheless, there are a few publications that deal with possible vaccines against the amoebae. In 2023, a research group from Mexico investigated the immunoprotected influence of two antigens. One of these antigens is the membrane protein MP2CL5 (Smp145). Both were injected intranasally into BALB/c mice. Cholera toxin (CT) served as an adjuvant. According to the study results, the antigens achieved a protection of 80–100%, respectively. In addition, a significant increase in T and B lymphocytes was observed. MP2CL5 is currently considered a promising candidate for a vaccine against *N*. *fowleri* [105]. In addition, research into *N*. *fowleri* also benefits from the new mRNA vaccines. mRNA vaccines against *N*. *fowleri* are already being discussed and in some cases have already been researched. In addition to feasibility, the location of the vaccination site is also part of these discussions. As a rule, the extremities are vaccinated: the arm or, in young children, in the leg. Intranasal vaccinations, i.e. vaccinations in the nose, have already been discussed for SARS-CoV-2 [106]. Since it is known that its tissue-specific exposure controls the immune system, intranasal vaccinations against *N*. *fowleri* are particularly interesting. After all, this is the first time the amoeba encounters the host tissue. Nevertheless, the development of a potent vaccine against *N*. *fowleri* remains questionable at the moment. Not least because of its rarity.

## Outlook and Conclusion

Because of its lethal consequences and speed, PAME is a serious disease. For this reason, the diagnosis can also be very frightening and stressful for patients. In conclusion, the prevalence of *N*. *fowleri* should be considered in the context of climate change. The prevalence of the amoeba is thought to change with increasing climate and is likely to become more important [107]. This assumption is supported by existing epidemiological data. Cases have been increasing since 2000 [108]. This also shows an increasing range in the geographical area as well as in the age of the patients. Simply put, *N*. *fowleri* is occurring more frequently in the elderly people in more countries. It should also be noted that in India, for example, cases occur more frequently that are not water-related, i.e., do not result from swimming. How exactly these cases arose is still unclear [108]. However, they point out that *N*. *fowleri* poses an increasing threat as climate changes and should be considered in the context of climate change. Its status as an NTD complicates important developments (both in science and in (health) policy). This could become a problem in the near future. The right course should therefore be set quickly.
